# Latent structure and measurement invariance of the Hospital Anxiety and Depression Scale in cancer outpatients

**DOI:** 10.1016/j.ijchp.2022.100315

**Published:** 2022-05-26

**Authors:** Elisabeth L. Zeilinger, Ingo W. Nader, Wolfgang Wiedermann, Mencia R. Gutierrez-Colosia, Matthias Unseld, Simone Lubowitzki, Markus Raderer, Philipp B. Staber, Peter Valent, Alexander Gaiger

**Affiliations:** a Division of Hematology and Hemostaseology, Department of Internal Medicine I, Medical University of Vienna, Vienna, Austria; b Division of Palliative Medicine, Department of Internal Medicine I, Medical University of Vienna, Vienna, Austria; c IT-Power Services GmbH, Vienna, Austria; d College of Education & Human Development and Missouri Prevention Science Institute, University of Missouri, Columbia, USA; e Department of Psychology, Universidad Loyola Andalucía, Sevilla, Spain; f Division of Oncology, Department of Internal Medicine I, Medical University of Vienna, Vienna, Austria; g Ludwig Boltzmann Institute for Hematology and Oncology, Medical University of Vienna, Austria

**Keywords:** Anxiety, Depression, Factor structure, Psychometric properties, Instrumental study

## Abstract

**Background/Objective:**

The aim of the present study was to compare competing psychometric models and analyze measurement invariance of the *Hospital Anxiety and Depression Scale* (HADS) in cancer outpatients.

**Method:**

The sample included 3,260 cancer outpatients. Latent structure of the HADS was analyzed using confirmatory factor analysis (CFA) with robust maximum likelihood estimation (MLR). Measurement invariance was tested for age, time of response, gender, and cancer type by comparing nested multigroup CFA models with parameter restrictions.

**Results:**

Except for the one-factor solutions, all models showed acceptable model fit and measurement invariance. The model with the best fit was the originally proposed two-factor model with exclusion of two items. The one-factor solutions showed inacceptable model fit and were not invariant for age and gender.

**Conclusions:**

The HADS has a robust two-factor structure in cancer outpatients. We recommend excluding item 7 and 10 when screening for anxiety and depression.

## Introduction

Depression and anxiety disorders are the most frequently diagnosed psychiatric complications in patients with cancer, with prevalence rates being two to four times higher than in people without a cancer diagnosis (Pilevarzadeh et al., 2019; Unseld et al., 2019). Psychiatric comorbidities greatly affect patient quality of life and treatment adherence, and negatively impact physical health outcomes and mortality (Gaiger et al., 2022; Unseld et al., 2021). Recognition and treatment of psychiatric disorders is crucial in multidisciplinary care (Pitman et al., 2018; Tsaras et al., 2018). Routine screening instruments are particularly valuable for early detection. In our study, we analyzed one of the most frequently used screening tools, the *Hospital Anxiety and Depression Scale* (HADS, Zigmond & Snaith, 1983).

The HADS was designed for routine screening in outpatient hospital settings and contains 14 items, seven each for anxiety and depression, rated on a four-point Likert scale. Validity studies in cancer patients showed favorable results in many languages (e.g. Annunziata et al., 2020; Hyland et al., 2019; Wondie et al., 2020). Due to its brevity and the availability in multiple languages, the HADS is frequently used in routine screening and international multicenter studies. However, analysis of the latent structure of the HADS has yielded some controversial results (Cosco et al., 2012), which also calls into question the scoring procedure to reliably screen for anxiety and depression.

Initially, the HADS was designed as a two-factor scale. Although not proposed by the authors of the HADS, the total score including all 14 items is frequently used as a measure of psychological distress. Some studies have also proposed and tested a variety of three-factor models with correlated or uncorrelated factors and a hierarchical or non-hierarchical structure (Caci et al., 2003; Dunbar et al., 2000; Friedman et al., 2001) following the tripartite theory, where anxiety and depression are characterized by both shared and unique features (Clark & Watson, 1991). The present study examines twelve models of the HADS as depicted in Table 1.

**Table 1:** Fit statistics for different models of the HADS.

Model	Original study			Model specifications			Present study results			
	Original sample	Original analysis	*n*	No. of factors	Factors and Items	Excluded items	*χ*^2^_scaled_	*df*	CFI_robust_^a^	RMSEA_robust_^b^
Zigmond and Snaith (1983)	Medical	-	100	2	Anxiety: 1, 3, 5, 7, 9, 11, 13 Depression: 2, 4, 6, 8, 10, 12, 14	-	714.1	76	0.958	0.058
Zigmond and Snaith (1983) only mandatory items	Medical	-	100	2	Anxiety: 1, 3, 5, 9 Depression: 2, 4, 6, 12	7, 8, 10, 11, 13, 14	154.5	19	0.986	0.053
Razavi et al. (1989)	Cancer in-patients	Exploratory	210	1	General distress: 1–14	-	2157.3	77	0.863	0.104
Moorey et al. (1991)	Cancer	Exploratory	568	2	Anxiety: 1, 3, 5, 9, 11, 13 Depression: 2, 4, 6, 7, 8, 10, 12, 14	-	696.4	76	0.960	0.057
Dunbar et al. (2000)c	Non-clinical	Confirmatory	2,547	3	Autonomic anxiety: 3, 9, 13 Negative affectivity: 1, 5, 7, 11 Anhedonic depression: 2, 4, 6, 8, 10, 12, 14	-	529.6	73	0.970	0.050
Friedman et al. (2001)	Depressed	Exploratory	2,669	3	Psychic anxiety: 3, 5, 9, 13 Psychomotor agitation: 1, 7, 11 Depression: 2, 4, 6, 8, 10, 12, 14	-	533.6	74	0.970	0.050
Caci et al. (2003)c	Healthy students	Confirmatory	195	3	Anxiety: 1, 3, 5, 9, 13 Restlessness: 7, 11, 14 Depression: 2, 4, 6, 8, 12	10	690.6	62	0.958	0.063
Emons et al. (2012)	Coronary heart disease	Exploratory and Mokken analysis	534	2	Anxiety: 1, 3, 5, 9, 13 Depression: 2, 4, 6, 8, 12	7, 10, 11, 14	300.4	34	0.978	0.055
Smith et al. (2006; one-factor solution)	Cancer patients	Rasch analysis	1,855	1	Distress: 1, 2, 3, 4, 5, 6, 7, 8, 9, 12, 13	10, 11, 14	1776.2	44	0.867	0.125
Smith et al. (2006; two-factor solution)	Cancer patients	Rasch analysis	1,855	2	Anxiety: 1, 3, 5, 7, 9, 13 Depression: 2, 4, 6, 8, 12, 14	10, 11	576.8	53	0.963	0.062
13-item model				2	Anxiety: 1, 3, 5, 9, 11, 13 Depression: 2, 4, 6, 8, 10, 12, 14	7	438.8	64	0.973	0.048
12-item model				2	Anxiety: 1, 3, 5, 9, 11, 13 Depression: 2, 4, 6, 8, 12, 14	7, 10	364.7	53	0.977	0.048

a CFI > 0.9 indicates an adequate model fit.

b classification of RMSEA: < 0.05 = good, < 0.08 = acceptable, > 0.08 = not acceptable.

c These models had to be constrained. Note. the best model fit indices are indicated in bold.

A systematic review and meta-CFA described the latent structure of the HADS as unstable due to variation in study results (Cosco et al., 2012; Norton et al., 2013). However, we hypothesize that the variability in results is influenced mainly by three aspects: (1) characteristics of the sample, (2) single problematic items, and (3) the statistical methods used for determining factor structure.

First, examining the characteristics of samples in individual studies, some samples seem to yield a reasonable stable factor structure of the HADS. In patients with various types of cancer, a two-factor structure was supported by the majority of studies in different languages (e.g. Hyland et al., 2019; Moorey et al., 1991; Muszbek et al., 2006). In contrast, in patients with heart disease, most studies supported a three-factor solution (e.g. Emons et al., 2012; Martin et al., 2008). Studies on outpatients (i.e., the original target population of the HADS), including cancer outpatients, also supported a two-factor structure of the HADS (e.g. Moorey et al., 1991; Muszbek et al., 2006; Wiriyakijja et al., 2020).

Second, across studies, some items have shown to be difficult to allocate to one of the two factors, depression or anxiety. Considering that the HADS contains 14 items only, having one or two items that do not measure the same latent construct can have a considerable effect on the stability of the factor structure. Item 7 of the initial anxiety factor (‘I can sit at ease and feel relaxed’) often showed similar loadings on both factors, anxiety and depression, or was part of a third factor related to ‘restlessness’ (Moorey et al., 1991; Muszbek et al., 2006; Nezlek et al., 2021). It was argued that this specific item can overlap with problems caused by a somatic illness, e.g. in persons with spinal cord injury (Woolrich et al., 2006) or coronary heart disease (Martin et al., 2008). Another problematic item found in previous research was item 10 (‘I have lost interest in my appearance’) of the depression factor (Caci et al., 2003; Emons et al., 2012; Smith et al., 2006).

The third aspect contributing to an unstable factor structure is the statistical methods used. HADS data are ordinally scaled and usually positively skewed. Therefore, the Maximum Likelihood (ML) method as the default estimator for CFA should not by applied. Some studies analyzing the latent structure of the HADS did account for ordinality and/or skewness of data. However, the majority did not, thus applying methods not suitable for the data quality of the HADS, leading to potentially controversial results.

Measurement invariance is an indispensable prerequisite for a scale used in heterogeneous populations like cancer patients. Measurement invariance assumes that the same latent dimensions are measured, and that items function the same way in different groups, e.g. according to gender or age. If a scale is invariant its results can reliably be compared between groups. There are different levels of measurement invariance with increasing restrictions in parameters: (1) Configurational invariance assumes that the same factor structure holds in all groups, (2) metric (weak) invariance assumes that factor loadings are identical between groups, i.e., in every group each item contributes to the construct in the same way, (3) scalar (strong) invariance assumes that loadings and intercepts are identical. This is the necessary prerequisite for an instrument to compare mean scores across groups. Measurement invariance of the HADS has hardly been tested in cancer patients. A comparison of German an Ethiopian patients found only metric invariance (Wondie et al., 2020).

Our study aims at determining the latent structure of the HADS in a large sample of cancer outpatients using analysis methods suitable for the data. We include theoretically and empirically derived models, and test for invariance according to age, gender, cancer type and time of response (cohort effect), which, to the best of our knowledge, has never been performed in a sample of cancer patients. Based on this analysis, we propose an optimal factor structure and scoring procedure for the HADS to more reliably screen for anxiety and depression in cancer outpatients.

## Method

### Participants

The final sample for statistical analysis included 3,260 cancer outpatients (50.7% women). Age ranged from 18 to 92 years with a mean age of 58.41 (*SD* = 14.58). Cancer diagnosis was available for a subsample of *n* = 2,562 (78.6%) participants. The most frequently diagnosed solid tumor was breast cancer (*n* = 385, 15.0%), followed by lung cancer (*n* = 369, 14.4%). Hematological cancer was diagnosed in *n* = 429 (16.7%) of patients. Table 2 summarizes sample characteristics.

**Table 2:** Socio-demographic and clinical characteristics of the sample.

Characteristic	*n*	%^a^
Gender		
Female	1,654	50.7
Male	1,606	49.3
Marital status		
Single/widowed/divorced	1,119	36.5
Married/partnered	1,946	63.5
Children^b^	2,072	73.6
Living area		
Rural	803	27.3
Urban	2,134	72.7
Highest educational level		
Lower secondary education	318	10.6
Upper secondary education	1,385	46.4
Postsecondary education	595	19.9
University/college	689	23.1
Monthly household income		
< 800 Euro	136	5.1
800 - 1,300 Euro	580	21.7
1,300 - 2,200 Euro	898	33.5
> 2,200 Euro	1,064	39.7
Employment		
Unemployed	293	9.9
Employed / Self-employed	1,240	41.8
Retired	1,436	48.3
Cancer type		
Hematological	429	16.7
Solid tumor	2,133	83.3
Breast	385	15
Lung	369	14.4
Soft tissue	219	8.5
Pancreas	183	7.1
Head and neck	169	6.6
Colon / rectum	167	6.5
Brain	119	4.6
Kidney / urinary tract / bladder	108	4.2
Stomach / oesophagus	99	3.9
Female genital organs	66	2.6
Prostate	50	2
Hepatobiliary	48	1.9
Testis	45	1.8
Thyroid	35	1.4
Malignant melanoma	24	0.9
Other solid	47	1.8

a For calculation of percentages missing values were excluded.

b Reflects the number and percentage of participants answering “yes” to this question. Note. N = 3,260.

### Instruments

Questionnaires included the HADS (Zigmond & Snaith, 1983) and a sociodemographic profile. The HADS is a 14-item screening instrument for anxiety (7 items) and depression (7 items). All items are rated on a 4-point Likert scale. Item numbers and wording are depicted in Table 3.

**Table 3:** Summary statistics for the HADS items by subscale.

Item content	Item number	*M*	*SD*	Skew^a^
Anxiety subscale				
I feel tense / wound up	1	1.01	0.79	0.68
I get a frightened feeling as if something awful is about to happen	3	1.21	1.01	0.22
Worrying thoughts go through my mind	5	1.01	0.91	0.6
I can sit at ease and feel relaxed	7	0.92	0.85	0.6
I get a frightened feeling / butterflies in the stomach	9	0.75	0.75	0.91
I feel restless as if I have to be on the move	11	0.97	0.83	0.54
I get sudden feelings of panic	13	0.48	0.71	1.52
Depression subscale				
I still enjoy the things I used to enjoy	2	0.85	0.88	0.84
I can laugh and see the sunny side of things	4	0.71	0.82	0.92
I feel cheerful	6	0.76	0.91	1.01
I feel as if I am slowed down	8	1.31	0.96	0.33
I have lost interest in my appearance	10	0.49	0.84	1.63
I look forward with enjoyment to things	12	0.92	0.91	0.75
I can enjoy a good book/radio/TV program	14	0.5	0.79	1.64

a Standard error of skewness in this sample = 0.04. Note. N = 3,260.

### Procedure

The present study was embedded in a larger ongoing research project performed at the outpatient clinic [*research site blinded for review*], aiming to assess psychosocial aspects in cancer patients. It was a single-center study. Data used in this study were collected from 2013 to 2021. In this time period, the HADS was used to screen for anxiety and depression at the outpatient clinic. Patients treated at the clinic were invited to participate upon the following inclusion criteria: (1) confirmed diagnosis of cancer, (2) age ≥ 18, (3) capacity to consent, and (4) sufficient German-language skills. A clinical psychologist or psychotherapist on site explained the research study. After informed consent, patients were handed out questionnaires to complete on their own during their waiting time. At any point within the study, patients had the opportunity to ask questions or withdraw from the study. The response rate was 78%. Patients cited lack of interest, insufficient time, or a desire not to be bothered with a study as reasons for not participating. The study was conducted in accordance with the International Conference on Harmonization E6 requirements for Good Clinical Practice outlined in the Declaration of Helsinki and approved by the institutional ethics committee of the research site (EC Nr: 473/2006; 1241/2021).

### Statistical methods

For analyzing the latent structure of the HADS, we used confirmatory factor analysis (CFA), which is usually estimated via a maximum likelihood (ML) approach. However, this estimator assumes normally distributed continuous data and is less suited for ordinal and potentially skewed data. Two possible alternatives are the robust ML estimation (MLR) and the weighted least squares mean and variance adjusted estimator (WLSMV; Muthén, 1984). MLR can be used with skewed distributions but assumes continuous data. WLSMV can be used with ordinal data but assumes normal distribution of the underlying latent dimension. We decided to use MLR, a maximum likelihood estimation with robust (Huber-White) standard errors and a scaled test statistic that is (asymptotically) equal to the Yuan-Bentler test statistic (Yuan & Bentler, 2000). This decision was based on the following reasons: (1) the latent dimensions anxiety and depression are not normally distributed in the population, thus basic assumptions of the WLSMV are violated. (2) Using MLR on the 4-point Likert scale of the HADS can lead to underestimated factor loadings (Rhemtulla et al., 2012). However, this potential bias is constant across all estimated models in our analysis and will not affect model comparisons. (3) MLR estimates allow for the use of ΔCFI, an effect size to judge measurement invariance, which is not possible for WLSMV (Sass et al., 2014). (4) MLR was shown to have better Type I error rates for the model tests (Li, 2014). We used robust CFI and robust RMSEA as fit indices.

Measurement invariance was tested by comparing nested multigroup CFA models with restrictions in parameters. Since Chi-Squared tests have been shown to be overly sensitive, especially in large samples (Cheung & Rensvold, 2002; Sass et al., 2014), we used ΔCFI which is less dependent on sample size and model complexity (Cheung & Rensvold, 2002). To evaluate measurement invariance, a commonly used criterion is a change in CFI by -0.01 (Putnick & Bornstein, 2016). This process is very well established for ML estimation and also valid for MLR estimation (Sass et al., 2014).

In total, twelve models were tested. These included ten models established by previous studies, and two newly suggested models. For designing the new models, we used the originally proposed structure and excluded one (item 7) or two items (item 7 and 10) that had been found problematic in a number of psychometric analyses of the HADS. All models and item allocations are depicted in Table 1.

We tested measurement invariance for four dichotomous grouping variables: age (< 60 years vs. ≥ 60 years), time of response (January 2, 2013 – August 16, 2016 vs. August 17, 2016 – May 25, 2021), gender (male vs. female), and cancer type (solid tumor vs. hematological cancer). Age and gender were examined to identify differences due to sociodemographic characteristics of the patients. Time of response provides insights into possible cohort effects. Because the type of physical illness may affect the presentation of psychiatric comorbidities, we also tested for differences by cancer type.

First, configurational invariance was established by fitting the models to each group individually. Second, metric (weak) invariance was established by fitting multigroup models with factor loadings constrained to be equal across groups. Third, scalar (strong) invariance was established by fitting multigroup models with factor loadings and intercepts constrained to be equal across groups. All analysis were performed in R (R Core Team, 2020) using the packages lavaan (Rosseel et al., 2021) and semTools (Jorgensen et al., 2021).

### Results

Descriptive statistics of HADS items are shown in Table 3. All items were positively skewed (values concentrated at the lower end of the scale), violating the distributional assumptions of the MLR estimation.

Model fit of all considered models is given in Table 1. Only the one-factor solutions had an unacceptable model fit, indicated by both CFI and RMSEA values. The two models that showed a good fit (RMSEA values < 0.05) were the ones with the original structure and exclusion of one or two items. All remaining models, including the original structure, showed acceptable CFI and RMSEA values. The best fitting model according to CFI and RMSEA was the original model after excluding items 7 and 10. Internal consistencies for this model were α = .828 for the anxiety and α = .867 for the depression factor.

**Figure 1:**
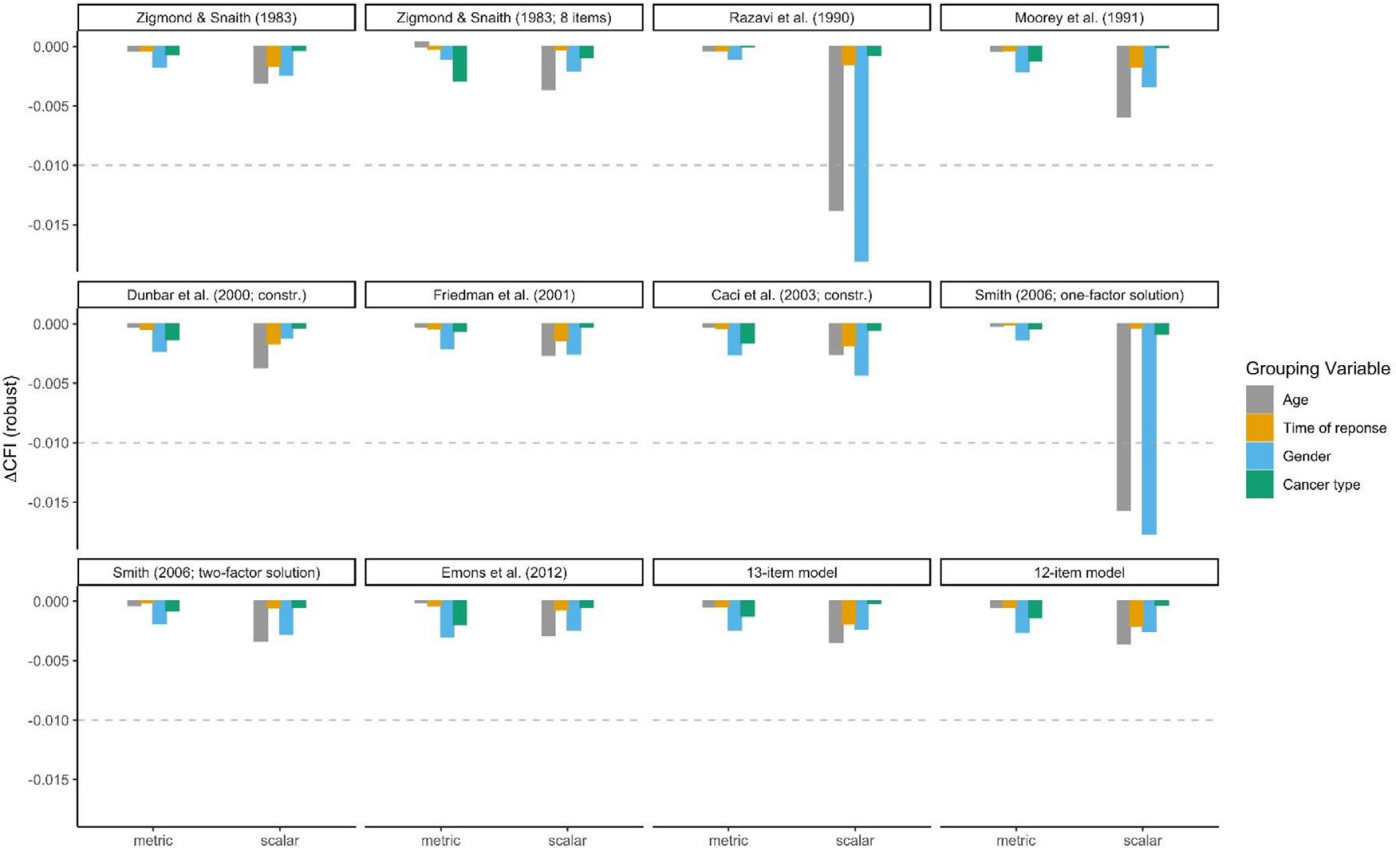
Measurement invariance for twelve models of the HADS according to age, time of response, gender, and cancer type. The figure shows measurement invariance of each of the twelve models tested. To achieve measurement invariance, ΔCFI should be > -0.01. This cut-off is indicated by dotted lines. If the bars plotted cross this line the model is not invariant for the respective grouping variable. Only the one-factor models were not invariant for age and gender. For all other models measurement invariance can be assumed.

Measurement invariance results for all models are presented in Figure. 1. Ten of the twelve models tested achieved scalar measurement invariance for all four covariates, i.e. age, time of response, gender, and cancer type. Only the one-factor solutions were not invariant according to age and gender. Table 4 details test results for the model with the best fit in the total sample. Further, the CFA estimation of the Dunbar three-factor model and the measurement invariance models of the Caci three-factor model initially yielded invalid solutions. The covariance matrix of latent variables was not positive definite for both models. Therefore, for the Dunbar model, the correlation between factors had to be constrained to a value smaller than 0.99. For the Caci model, the correlation between factors had to be constrained to a value of smaller than 0.94. The necessity of these constrains indicate that two of the three factors in these models measure the same construct.

**Table 4:** Measurement invariance of the 12-item model with best overall model fit.

Model	Group	Constraint	*χ*^2^_scaled_	*df*	CFI_robust_	ΔCFI_robust_
12-item model (original excluding item 7 & 10)	Age	G1: < 60 (n=1605)	193.4	53	0.979	
		G2: ≥ 60 (n=1655)	237.5	53	0.973	
		Configurational	431.0	106	0.976	
		Metric	449.9	116	0.975	-0.001
		Scalar	515.2	126	0.972	-0.004
	Time of response	G1: ≤ 2016-08-16 (n=1630)	231.9	53	0.973	
		G2: > 2016-08-16 (n=1630)	181.3	53	0.981	
		Configurational	412.7	106	0.977	
		Metric	431.1	116	0.977	0.000
		Scalar	474.6	126	0.975	-0.002
	Gender	G1: men (n=1606)	223.6	53	0.974	
		G2: women (n=1654)	207.6	53	0.978	
		Configurational	431.0	106	0.976	
		Metric	477.4	116	0.973	-0.003
		Scalar	527.9	126	0.971	-0.003
	Cancer type	G1: solid (n=988)	142.0	53	0.977	
		G2: hematological (n=338)	104.3	53	0.969	
		Configurational	248.8	106	0.974	
		Metric	266.5	116	0.973	-0.001
		Scalar	280.3	126	0.973	0.000

Note. To achieve measurement invariance, ΔCFI should be > -0.01.

## Discussion

The present study analyzed the factor structure of the HADS in cancer outpatients and examined measurement invariance of twelve different model specifications according to age, time of response, gender, and cancer type. The best fitting model was a 12-item model with the originally proposed item allocation and excluding items 7 and 10. Furthermore, the initially proposed two-factor structure with 14 items (Zigmond & Snaith, 1983) had an acceptable model fit and can thus also be used in cancer outpatients.

The one factor models showed unacceptable model fits and were not invariant for gender and age (recall that a one-factor solution was not initially proposed for the HADS). The first study suggesting a one-factor solution was conducted on a small sample of 228 French cancer in-patients (Razavi et al., 1989). A probabilistic study using Rasch analysis also identified one factor, but only after exclusion of three unscalable items (Smith et al., 2006). Other studies testing the one-factor solution of the HADS also found an unacceptable model fit (Albatineh et al., 2021). Although hierarchical models with a higher order distress factor may justify the computation of a total score, these models are not practical in a clinical setting because scoring can be overly complicated (Martin et al., 2008) and specific diagnostic information about whether a patient has symptoms of anxiety, depression or both is lost when using a total score (Emons et al., 2012).

Two of the three-factor models we tested could only be analyzed when adding constraints to avoid latent factor correlations larger than 1. As already discussed by Caci and colleagues (2003), the first analysis of the tripartite model by Dunbar and colleagues (2000) also produced high correlations between two of the three factors in the model. Overall, this indicates that two of the three factors measure the same dimension and should not be regarded or scored as distinct constructs. The tripartite theory of anxiety and depression (Clark & Watson, 1991) has its merits, but may not be applicable to the HADS. Most importantly, the HADS was not designed based on this model. Therefore, it may not be possible to reproduce the tripartite model with the 14 HADS items.

Our results support the use of the HADS in cancer outpatients and contradict the voices criticizing the HADS as unstable. Previous studies did not comprehensively consider three important aspects in their unfavorable evaluation of the HADS: (1) characteristics of the sample, (2) single problematic items, and (3) statistical methods used for determining the factor structure. A meta CFA including 21 studies with various patient and community samples found a bifactor solution of the HADS, with a strong general distress factor and two uncorrelated anxiety and depression factors (Norton et al., 2013). However, this study applied analysis methods that did not account for skewness of the data, which may have biased results. Furthermore, studies with a number of different patient- and community samples were included. Since there are indications that the factor structure of the HADS may vary in samples with different characteristics or with different physical conditions, like heart disease or cancer, the results may not reflect the structure of either the included samples.

One point of criticism voiced about the HADS is the omission of important somatic symptoms of depression, such as changes in appetite and sleep disturbance (Coyne & van Sonderen, 2012). As in cancer patients, these symptoms can easily be associated with the illness and/or treatment of cancer, omitting these aspects in screening for depression may be a strong advantage of the HADS in this population, as well as in other patient groups.

Zigmond and Snaith (1983) initially introduced the HADS as a scale with eight mandatory items (1, 3, 5, 9 for anxiety, and 2, 4, 6, 12 for depression) and six additional items. Various previous studies suggested omitting unscalable items of the HADS to increase reliability of the two constructs (Caci et al., 2003; Emons et al., 2012; Martin et al., 2008). Our study data indicates that, for cancer outpatients, items 7 and 10 should be excluded when screening for anxiety and depression.

Assessing psychiatric comorbidities in cancer patients using well-evaluated instruments like the HADS or the Brief Symptom Inventory (Calderon et al., 2020) is crucial for providing appropriate psychosocial support. Given the high prevalence of psychiatric comorbidities in cancer patients (Unseld et al., 2019), treatment programs should be enforced (Ichikura et al., 2020), especially in underserved patient groups including patients with low socioeconomic status (Zeilinger et al., 2022). However, there are also well-evaluated tools for assessing positive mental health aspects, including resilience (Alarcón et al., 2020) and growth (Oliveira et al., 2021). These should receive additionally consideration in the context of holistic, personalized cancer care.

## Limitations

We only tested models suitable for routine clinical practice, thus allowing a straightforward scoring procedure. We did not include hierarchical models in our analysis, because they have to be scored using a sophisticated scoring algorithm which contradicts the intended use of the HADS and is not feasible in clinical practice. Furthermore, for cancer type, we only compared patients with hematological malignancies and solid tumors. No further comparisons between different cancer entities, e.g. breast cancer or lung cancer, were conducted. As a single-center German-language study, our results need to be validated in other cancer-outpatient samples and other languages. However, the large sample size of 3,260 people in the present study supports the reliability and robustness of our results.

## Conclusions

The HADS has a stable two-factor structure in cancer outpatients. Even though the initially proposed structure (Zigmond & Snaith, 1983) can be applied, we recommend excluding items 7 and 10, thus reducing the HADS to twelve items to more reliably screen for anxiety and depression.

## Funding

This research did not receive any specific grant from funding agencies in the public, commercial, or not-for-profit sectors.

## Declaration of Competing Interest

None.
